# Supplementary figures and images for: The small RNA locus map for Chlamydomonas reinhardtii
Source: PLoS One. 2020 Nov 19;15(11):e0242516. doi: 10.1371/journal.pone.0242516 (PMC7676726; doi:10.1371/journal.pone.0242516)

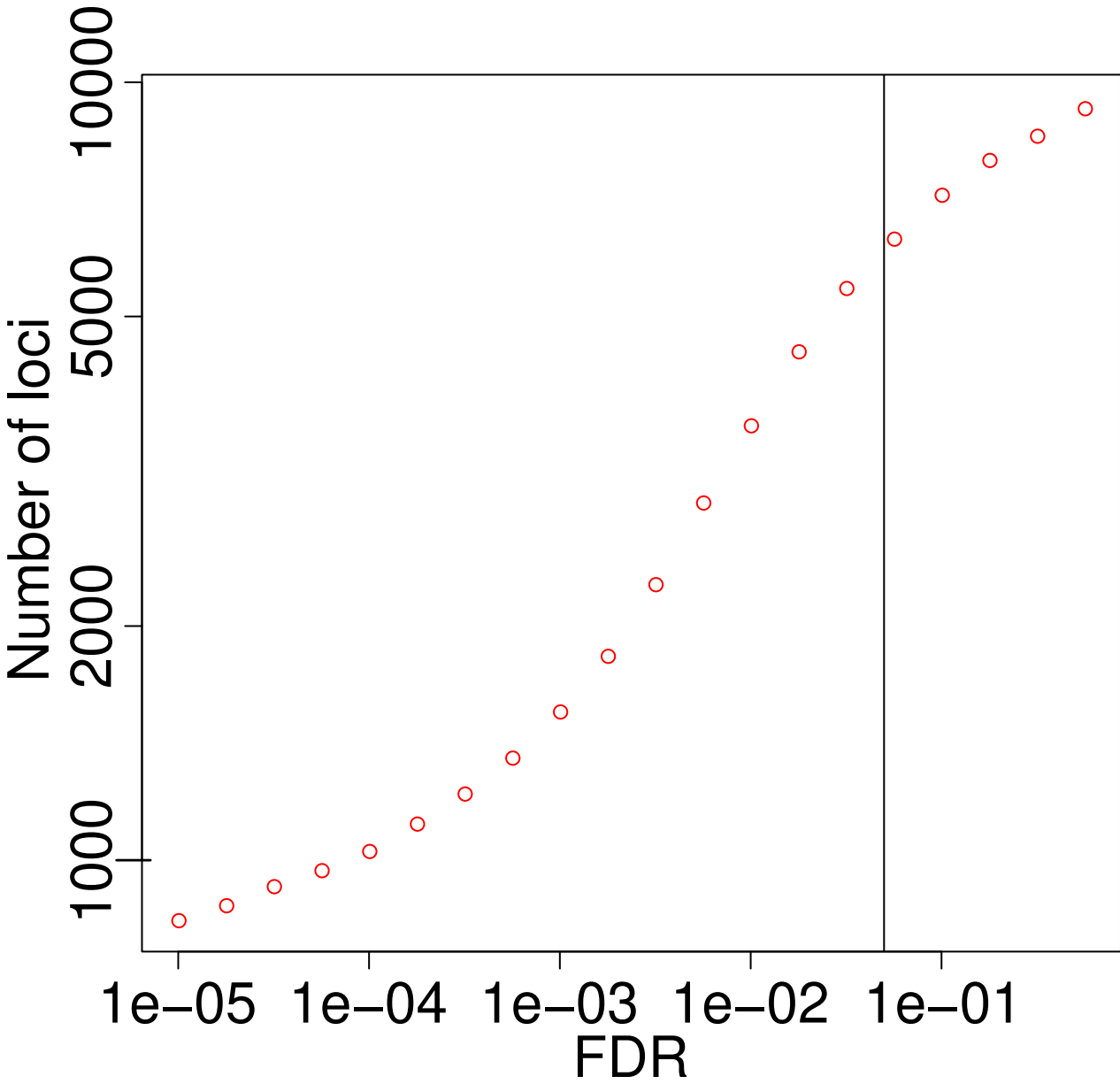

Supplement: S1 Fig — Plot of the number of loci for different FDR levels. Vertical black line corresponds to cutoff used (FDR = 0.05). (PDF) [file pone.0242516.s001.pdf]

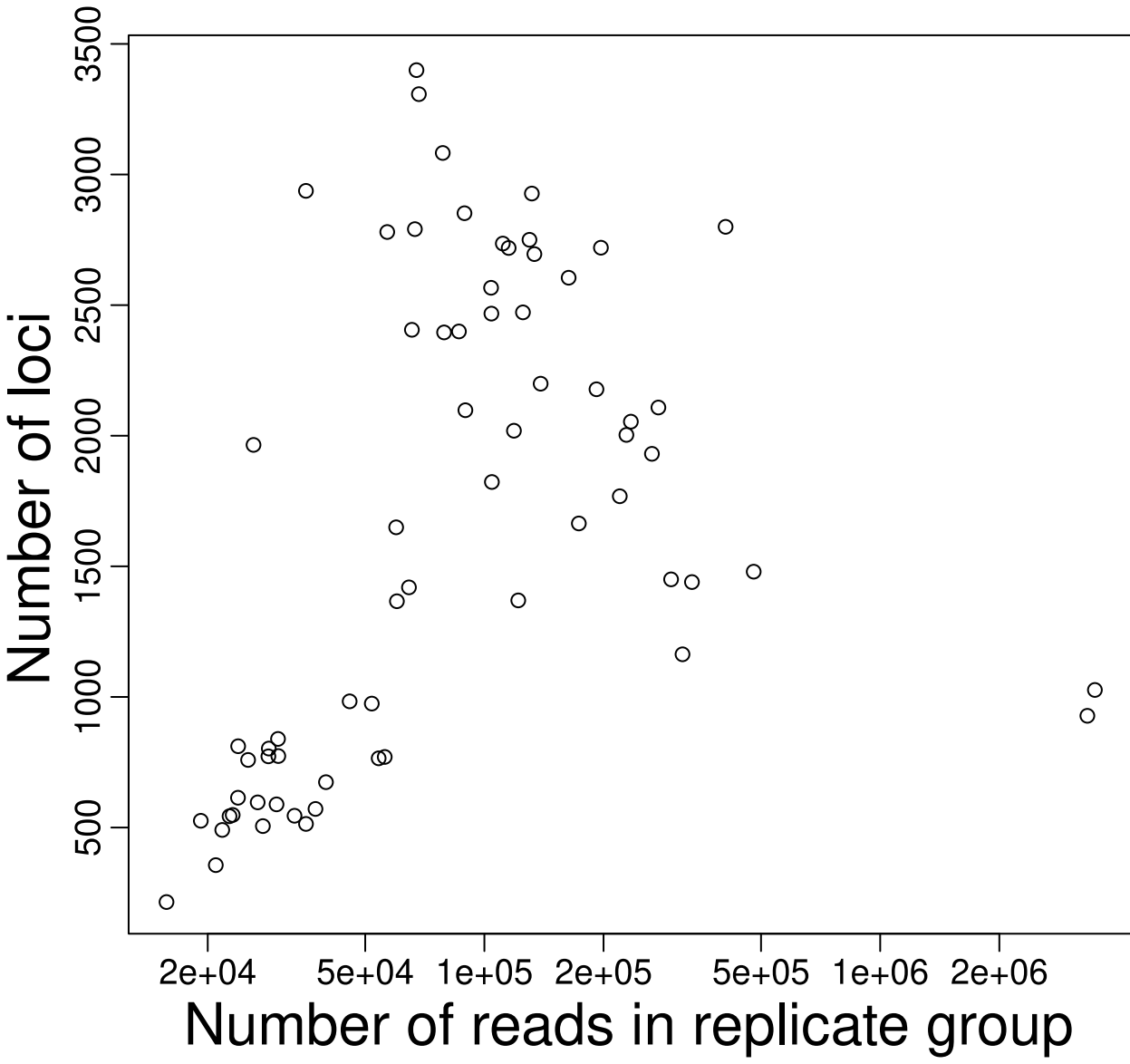

Supplement: S2 Fig — Number of loci discovered per replicate group plotted against library size. (PDF) [file pone.0242516.s002.pdf]

Total loci discovered

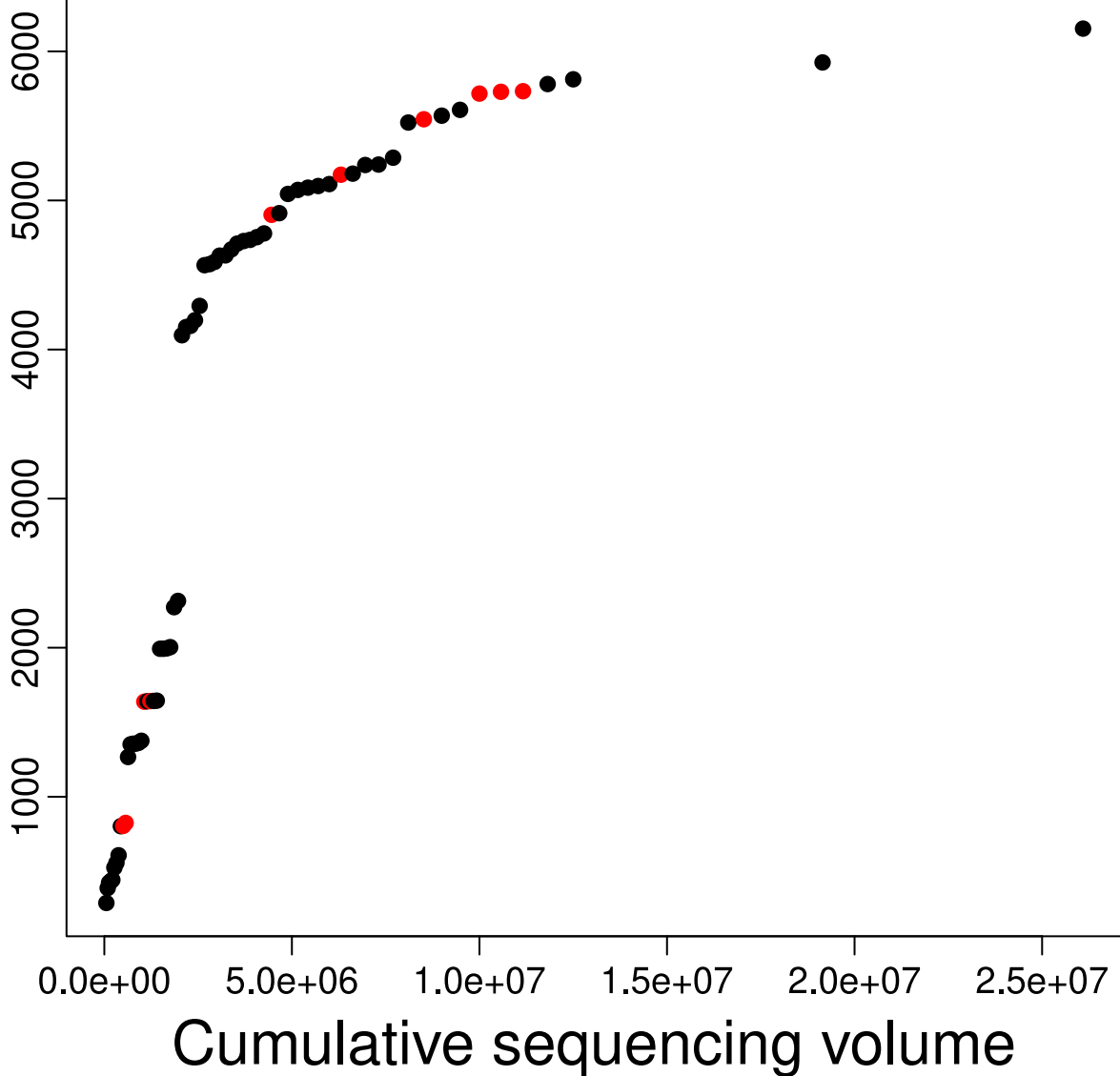

Supplement: S3 Fig — Scatter-plot of number of loci discovered as cumulative sequencing depth increases. Red dots represent WT libraries. (PDF) [file pone.0242516.s003.pdf]

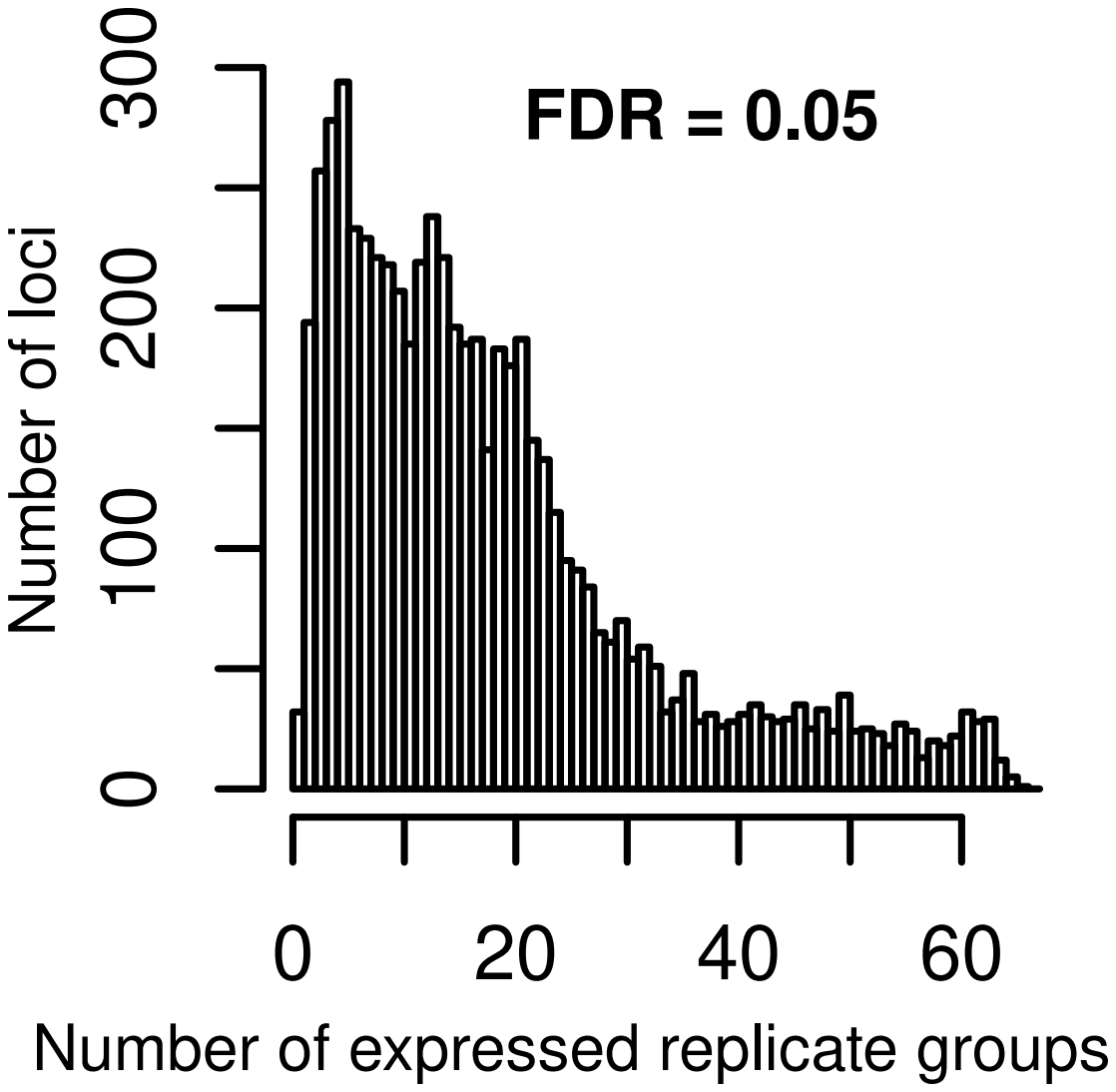

Supplement: S4 Fig — Number of loci expressed in a given number of replicate groups. (PDF) [file pone.0242516.s004.pdf]

# Locus size

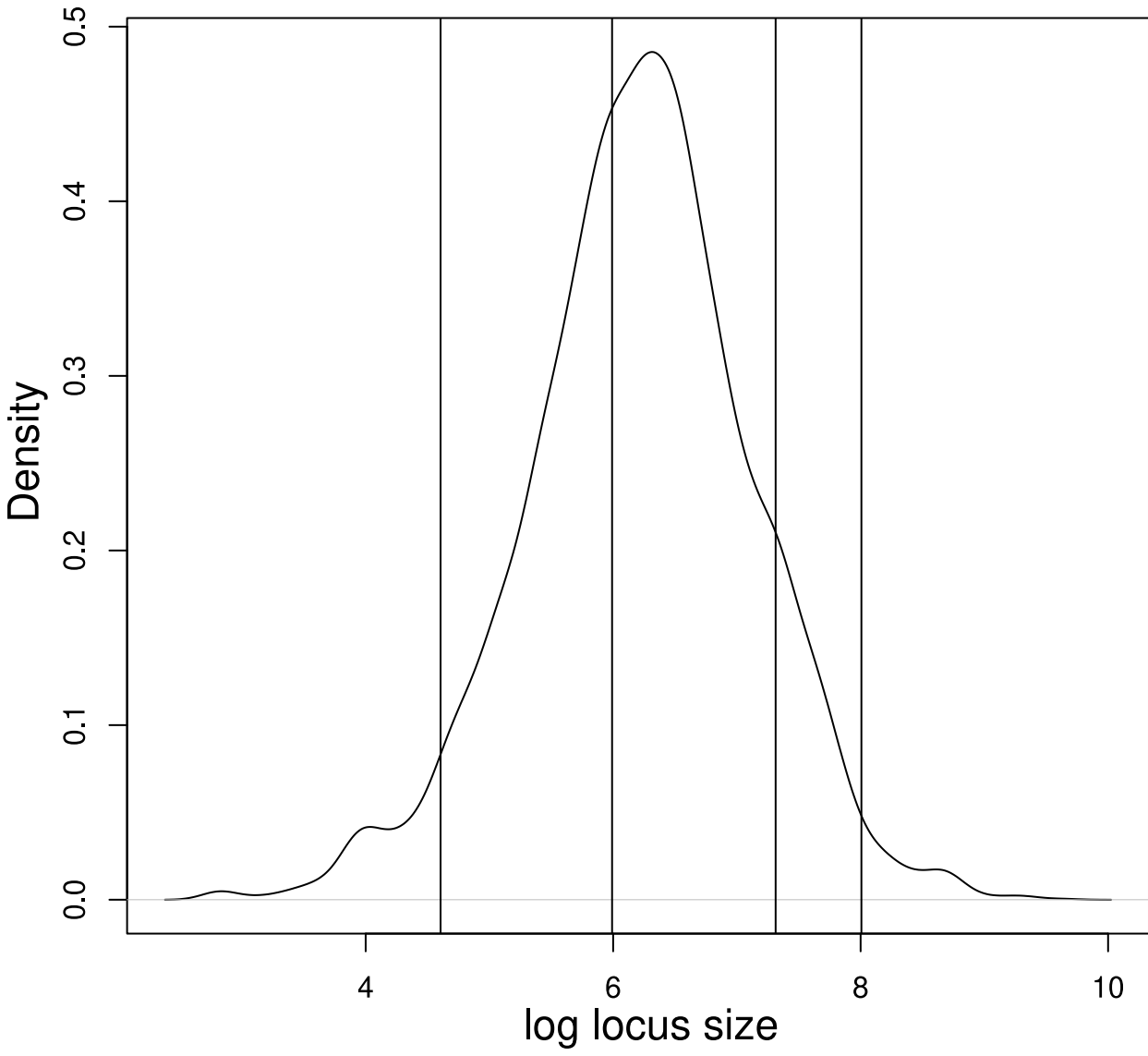

Supplement: S5 Fig — Density plot used to determine cut-offs for locus classification according to locus size. The vertical lines indicate the cut-offs used to classify the loci into discrete classes. (PDF) [file pone.0242516.s005.pdf]

# Repetitiveness

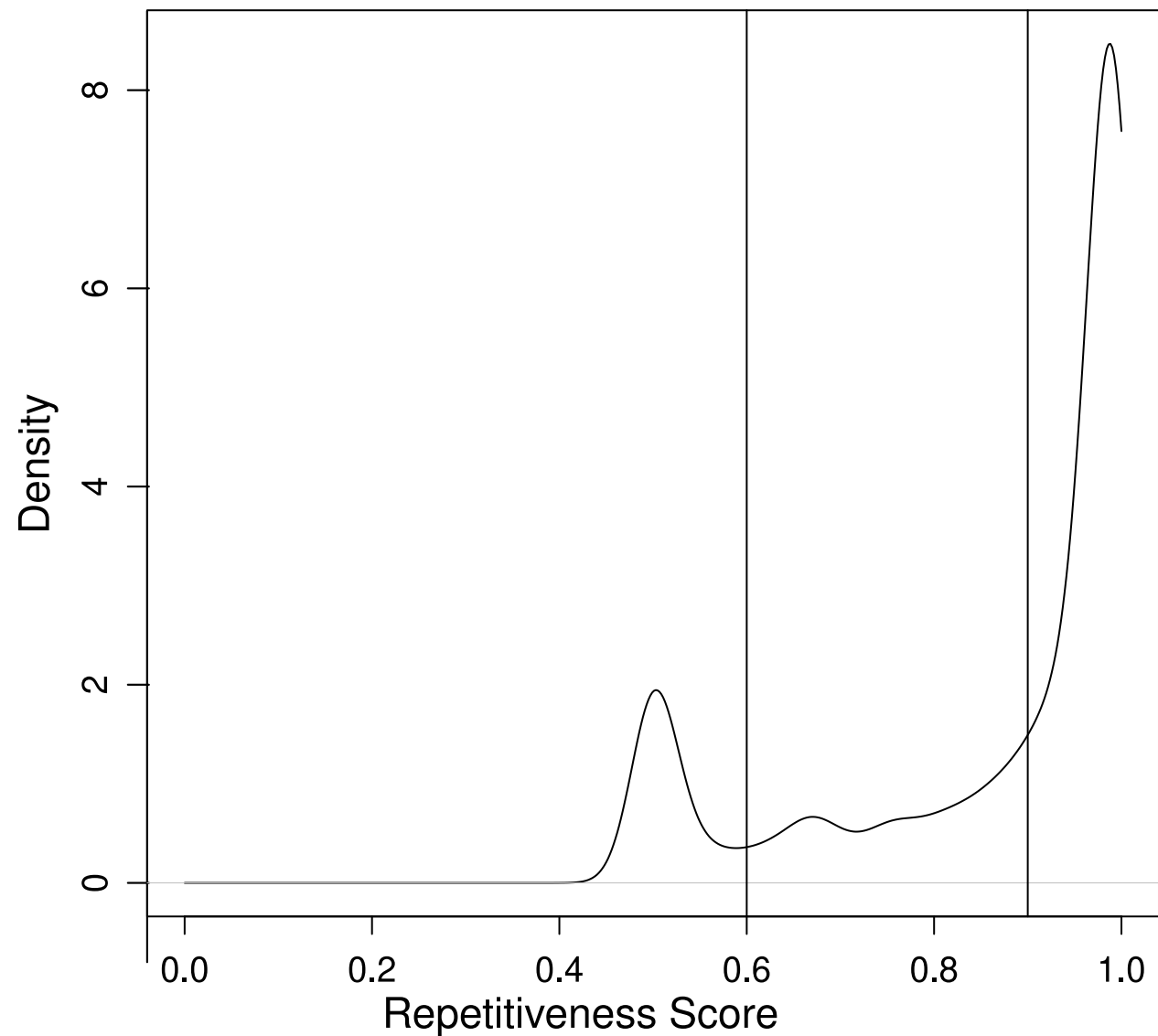

Supplement: S6 Fig — Density plot used to determine cut-offs for locus classification according to repetitiveness score. The vertical lines indicate the cut-offs used to classify the loci into discrete classes. (PDF) [file pone.0242516.s006.pdf]

# Strand bias

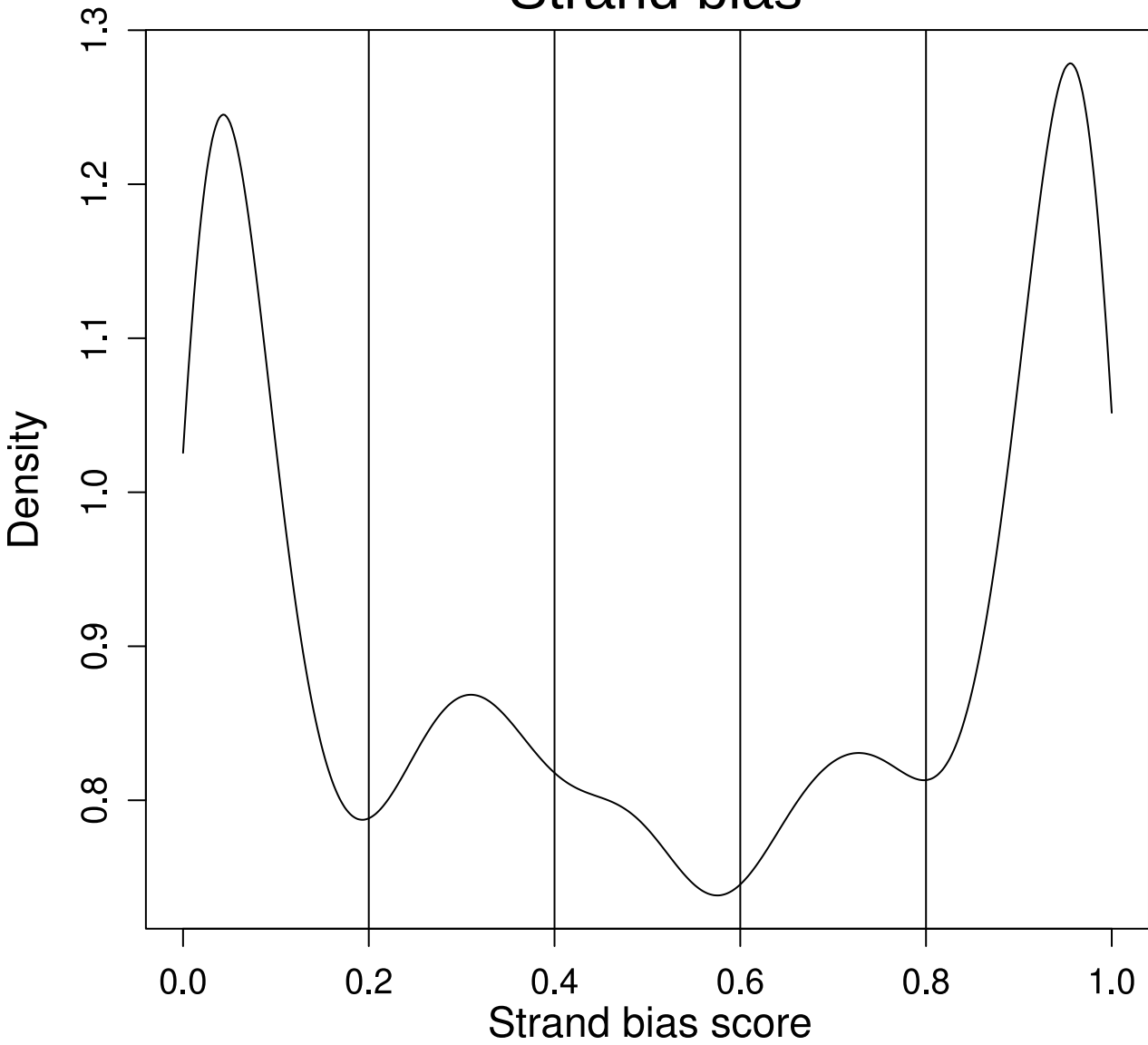

Supplement: S7 Fig — Density plot used to determine cut-offs for locus classification according to strand bias score. The vertical lines indicate the cut-offs used to classify the loci into discrete classes. (PDF) [file pone.0242516.s007.pdf]

# Phasing

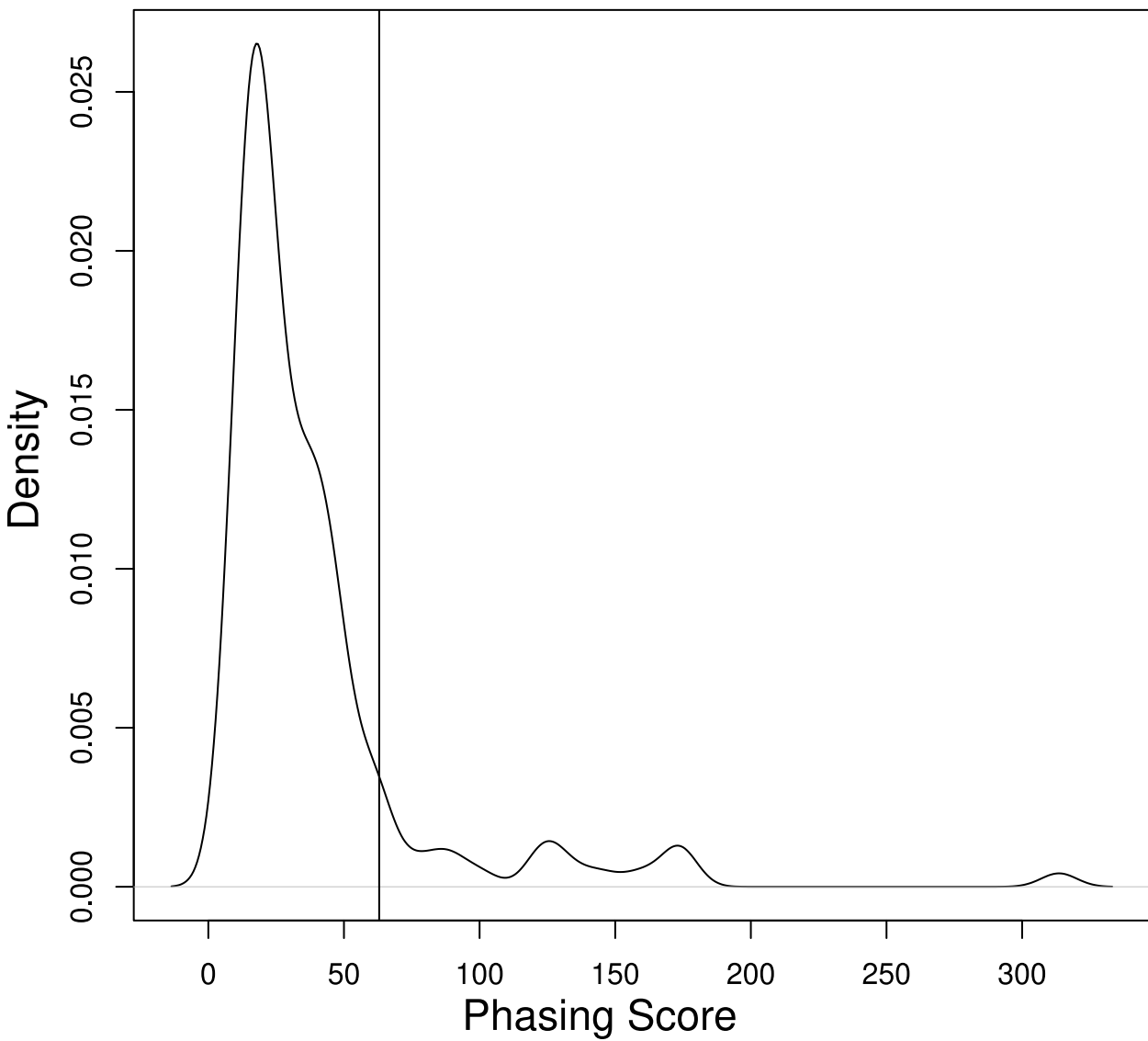

Supplement: S8 Fig — Density plot used to determine cut-off for locus classification according to phasing score. The vertical line indicates the cut-off used to classify the loci into two discrete classes. (PDF) [file pone.0242516.s008.pdf]

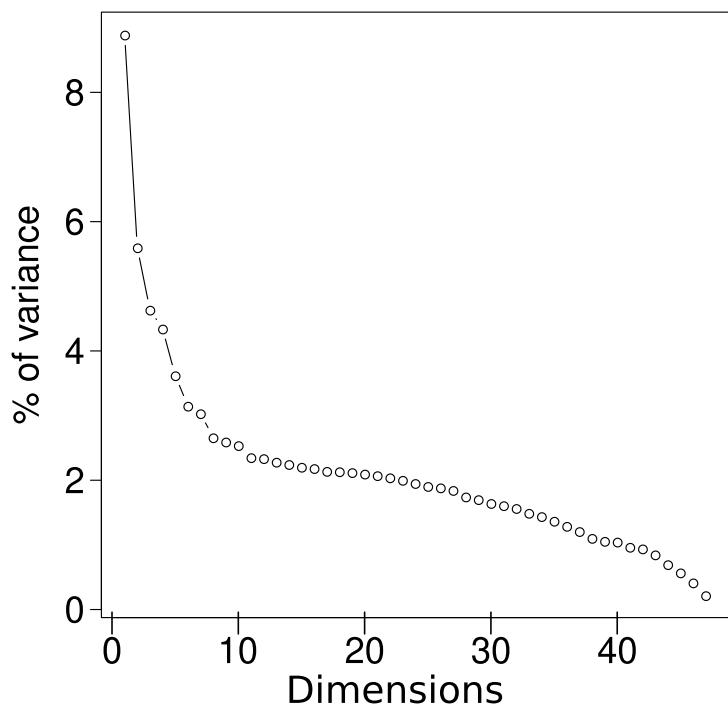

Supplement: S9 Fig — Ranked percentage (y-axis) of variance explained by each dimension (x-axis) of the MCA transformed data. (PDF) [file pone.0242516.s009.pdf]

Dimensions of MCA used

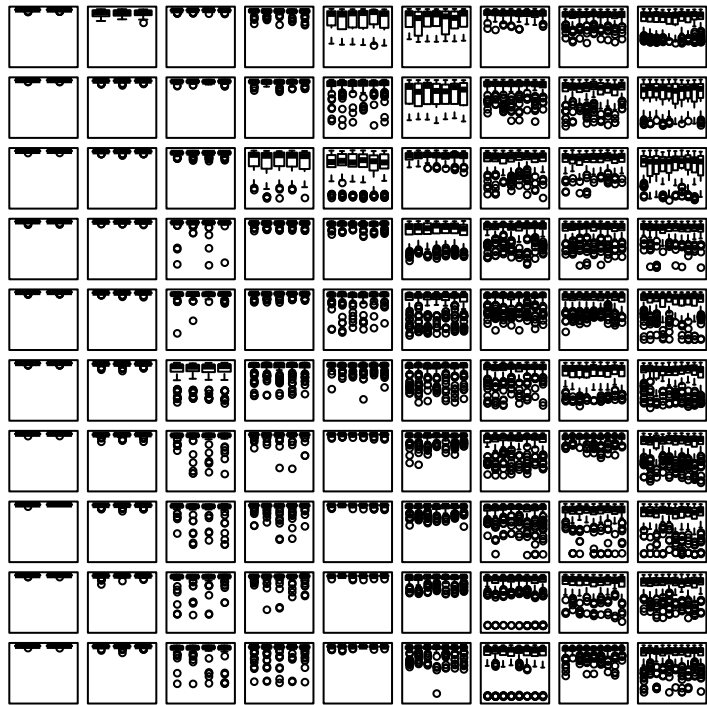

$k$

Supplement: S10 Fig — Stability of the cluster results (under bootstrapped sampling) achieved for all combinations of dimension selection from 1-8 and all numbers of clusters from 2-10. The y-axis for small plot indicates the proportion of loci which retained their original cluster assignments. The boxplots show the distribution of results after multiple iterations of the bootstrapped sampling and clustering. The x-axis corresponds to cluster result running k-means with (k) clusters. (PDF) [file pone.0242516.s010.pdf]

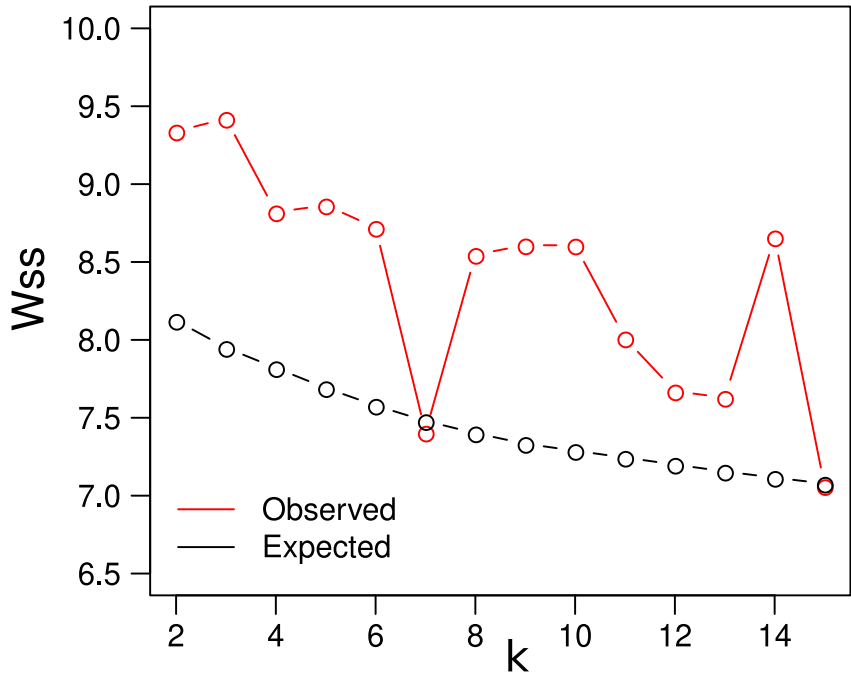

Supplement: S11 Fig — The x-axis corresponds to cluster result running k-means with (k) clusters. (PDF) [file pone.0242516.s011.pdf]

## NMI: TE superfamily

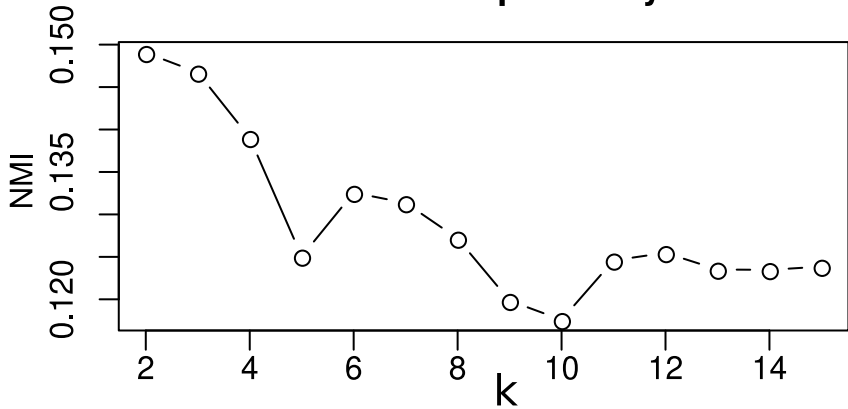

Supplement: S12 Fig — The x-axis corresponds to cluster result running k-means with (k) clusters. (PDF) [file pone.0242516.s012.pdf]

## NMI: Annotation features

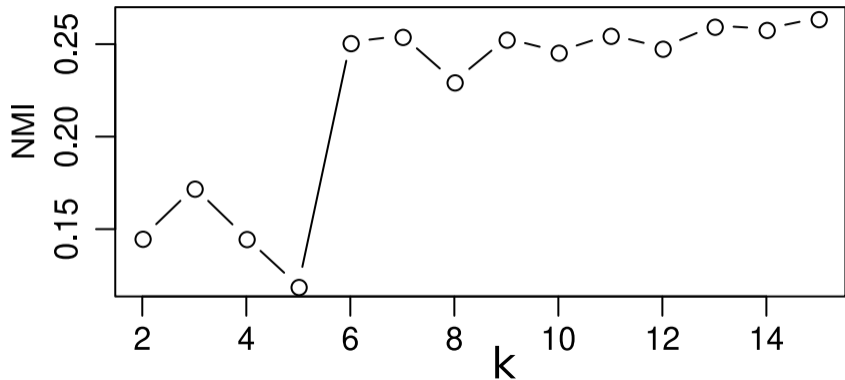

Supplement: S13 Fig — The x-axis corresponds to cluster result running k-means with (k) clusters. (PDF) [file pone.0242516.s013.pdf]

**FDR = 0.1**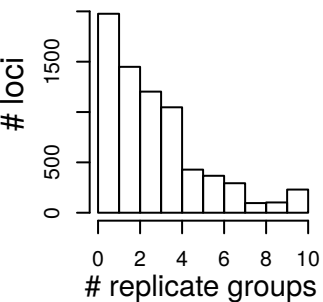**FDR = 0.05**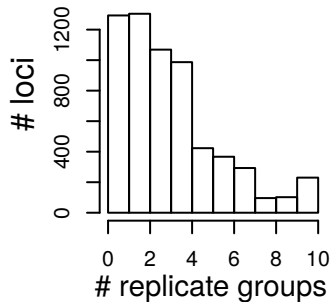**FDR = 0.01**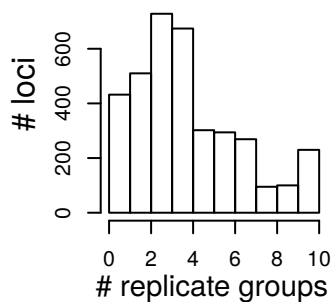**FDR = 0.001**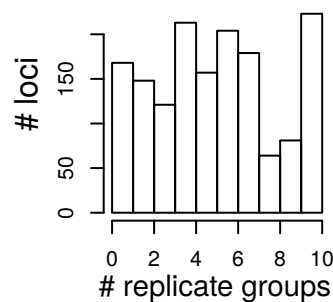**FDR = 1e-04**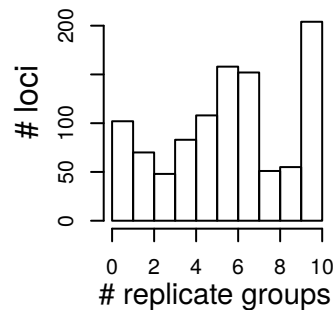**FDR = 0.1**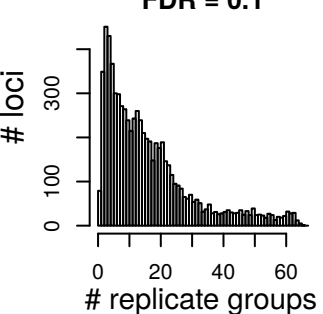**FDR = 0.05**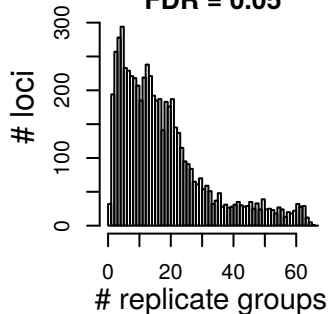**FDR = 0.01**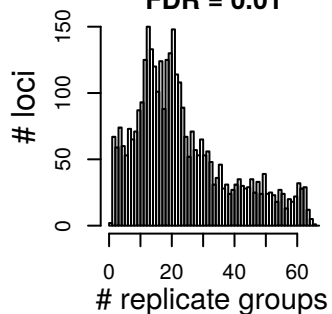**FDR = 0.001**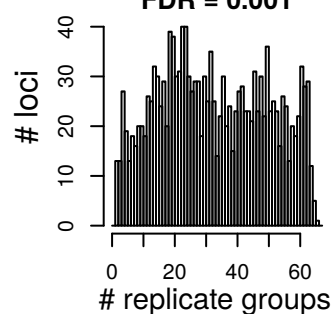**FDR = 1e-04**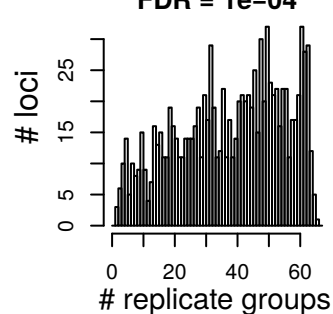**FDR = 0.1**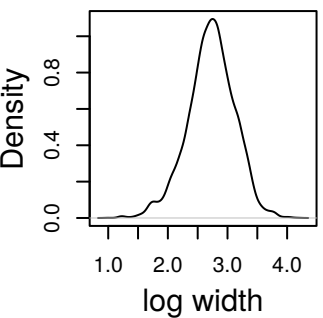**FDR = 0.05**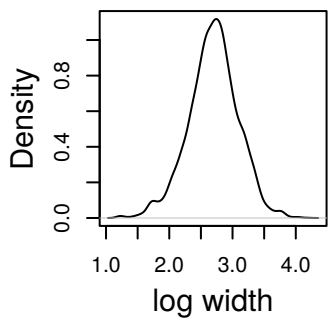**FDR = 0.01**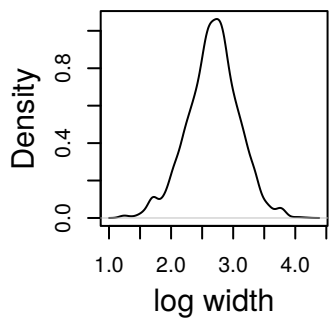**FDR = 0.001**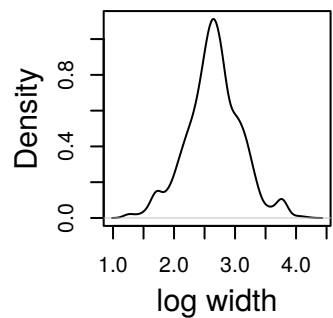**FDR = 1e-04**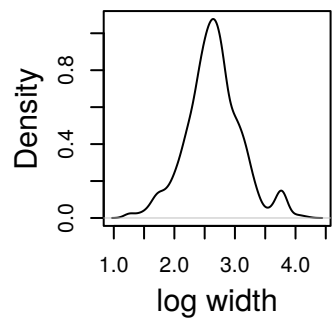

Supplement: S14 Fig — The top and middle rows show the frequency (y-axis) of loci found in a given number of replicate groups (x-axis) for the 10 wild type control replicate groups (top row) and all replicate groups (middle row). The bottom row shows locus size density distributions with the log of locus width on the x axis. (PDF) [file pone.0242516.s014.pdf]

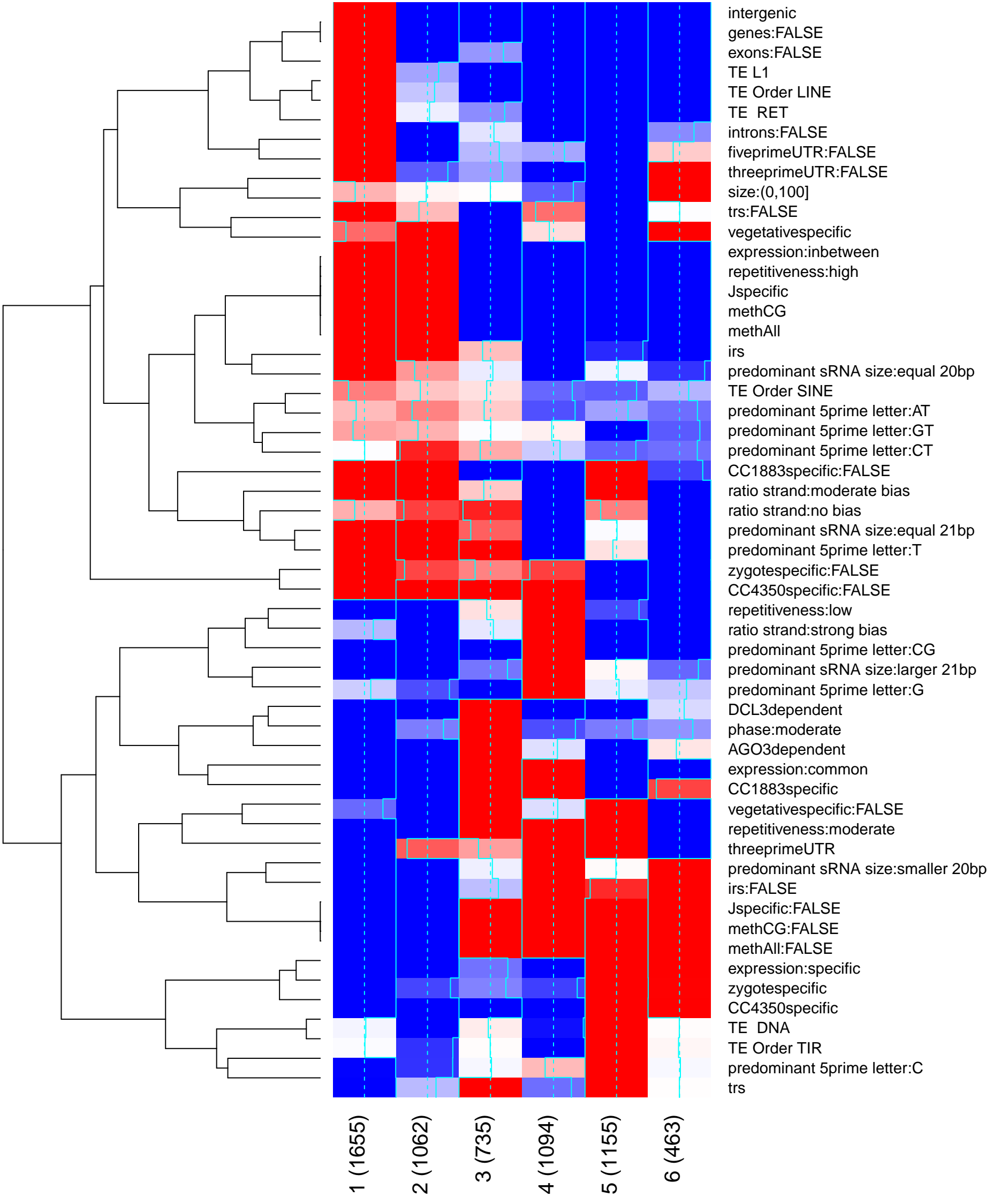

Supplement: S15 Fig — Red colours indicate association while blue colours represent disassociation. The size of the clusters is shown in brackets along the x axis. (PDF) [file pone.0242516.s015.pdf]

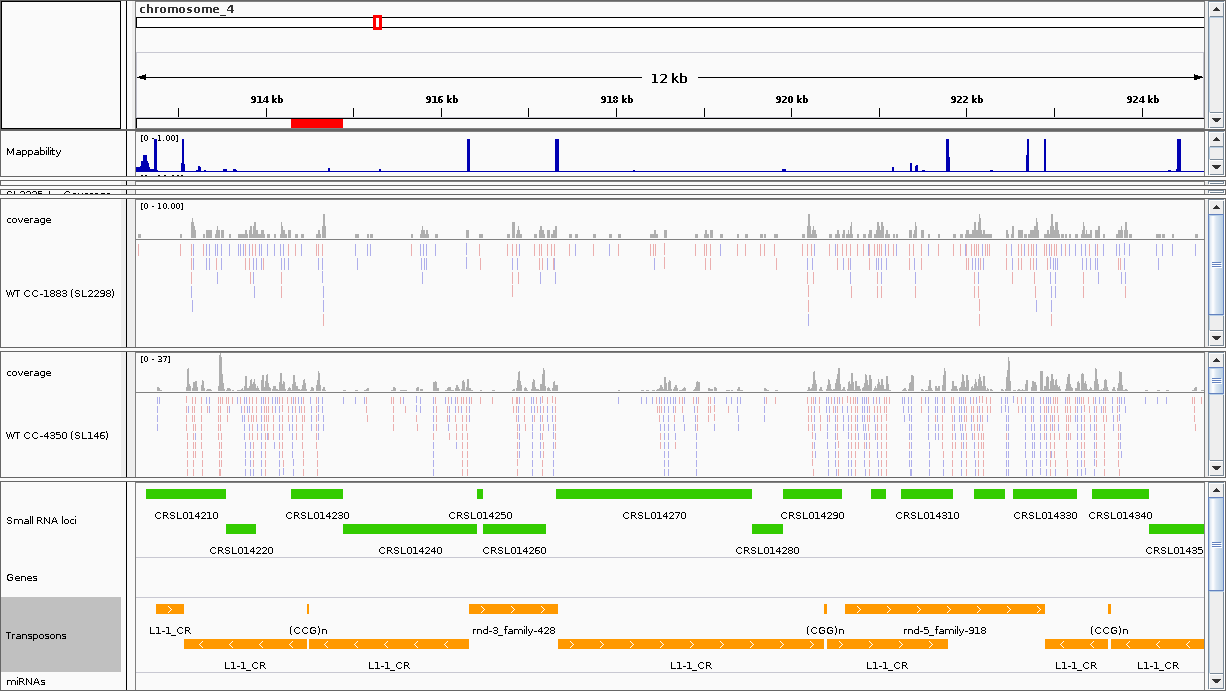

Supplement: S16 Fig — Tracks are annotated as in Fig 2. (PNG) [file pone.0242516.s016.png]

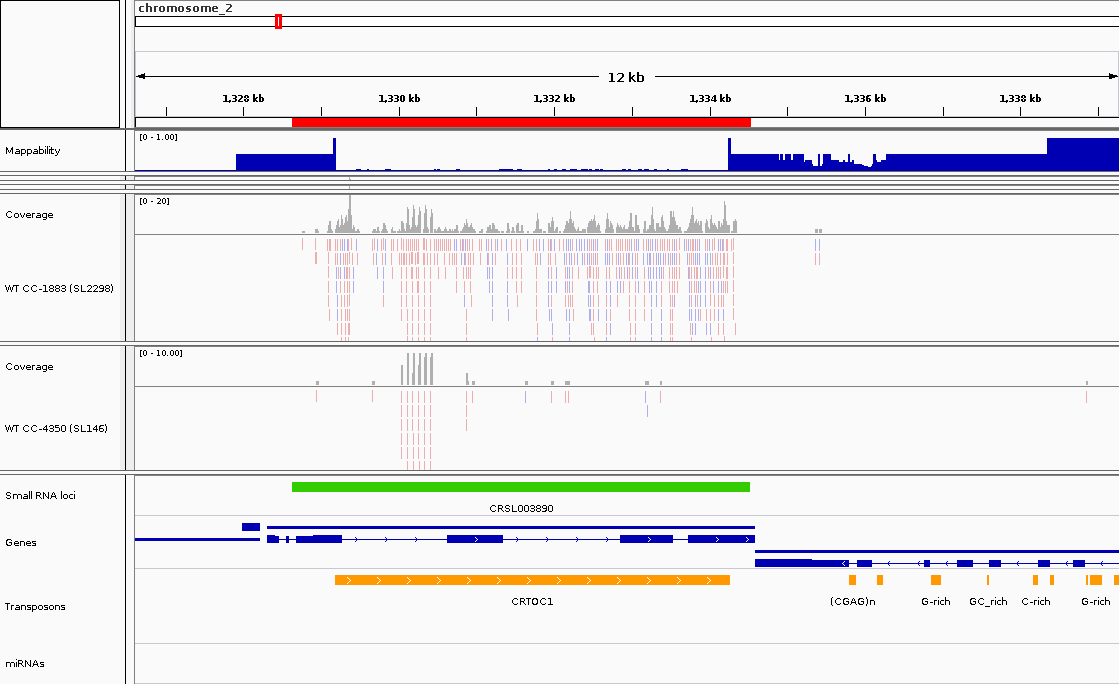

Supplement: S17 Fig — Tracks are annotated as in Fig 2. (PNG) [file pone.0242516.s017.png]

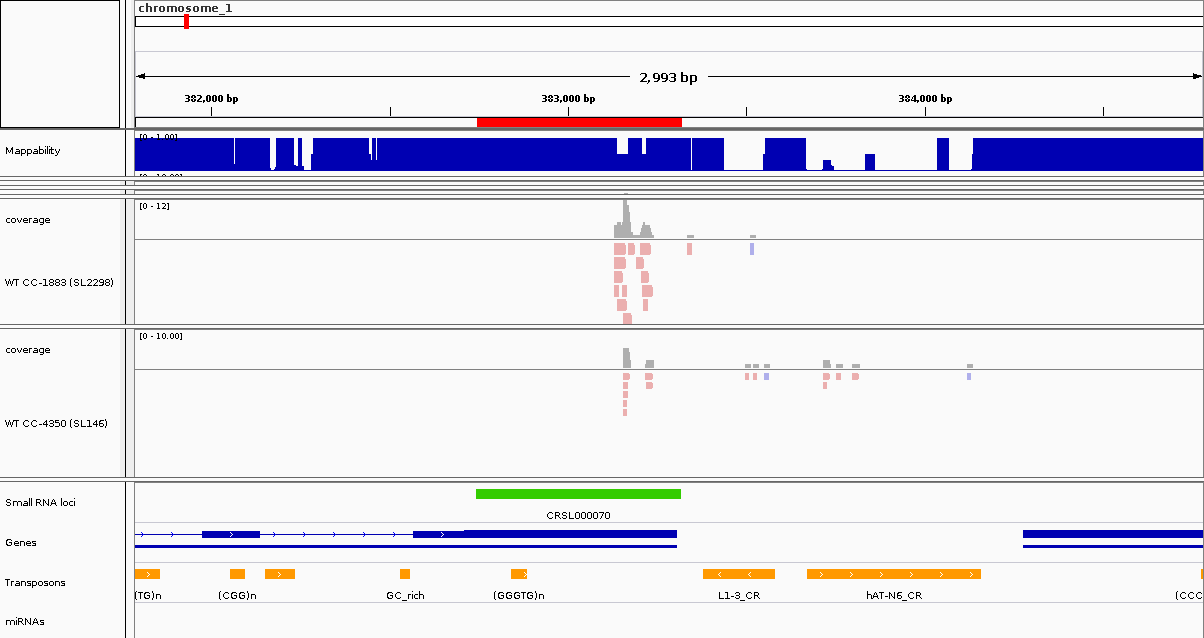

Supplement: S18 Fig — Tracks are annotated as in Fig 2. (PNG) [file pone.0242516.s018.png]

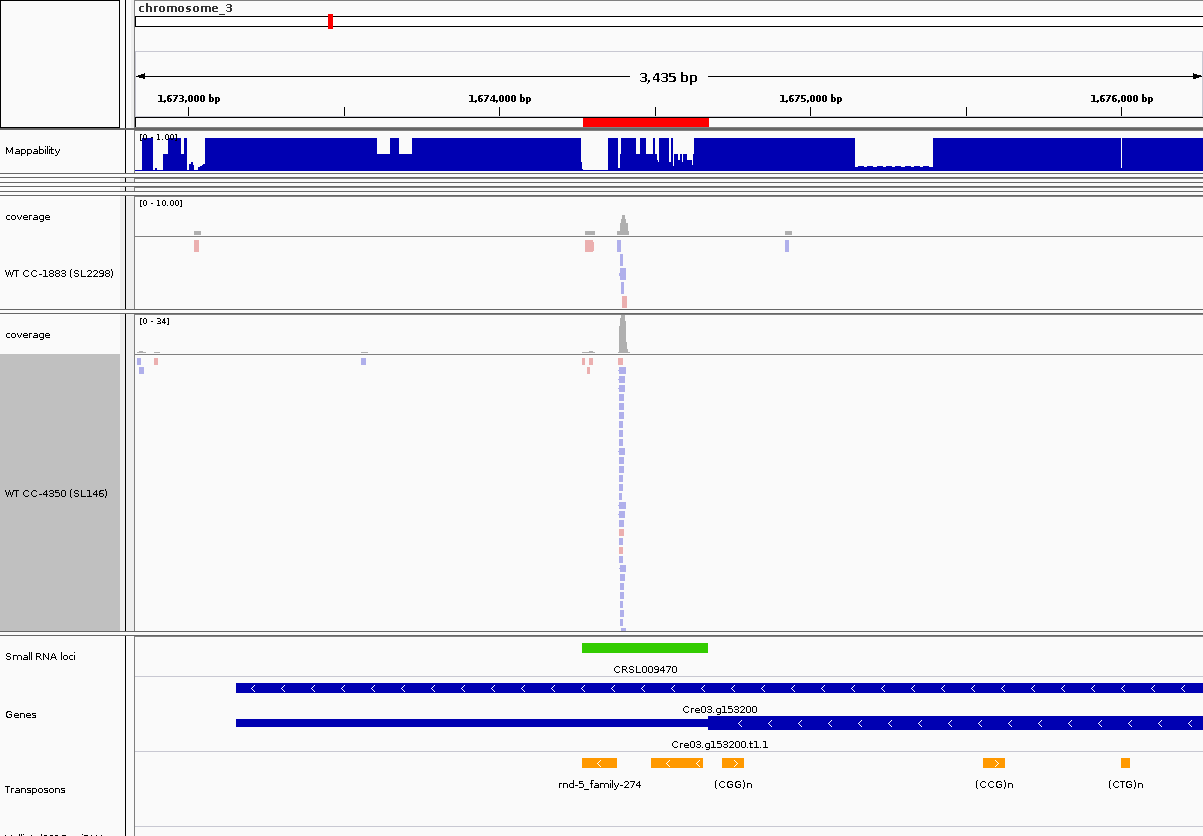

Supplement: S19 Fig — Tracks are annotated as in Fig 2. (PNG) [file pone.0242516.s019.png]

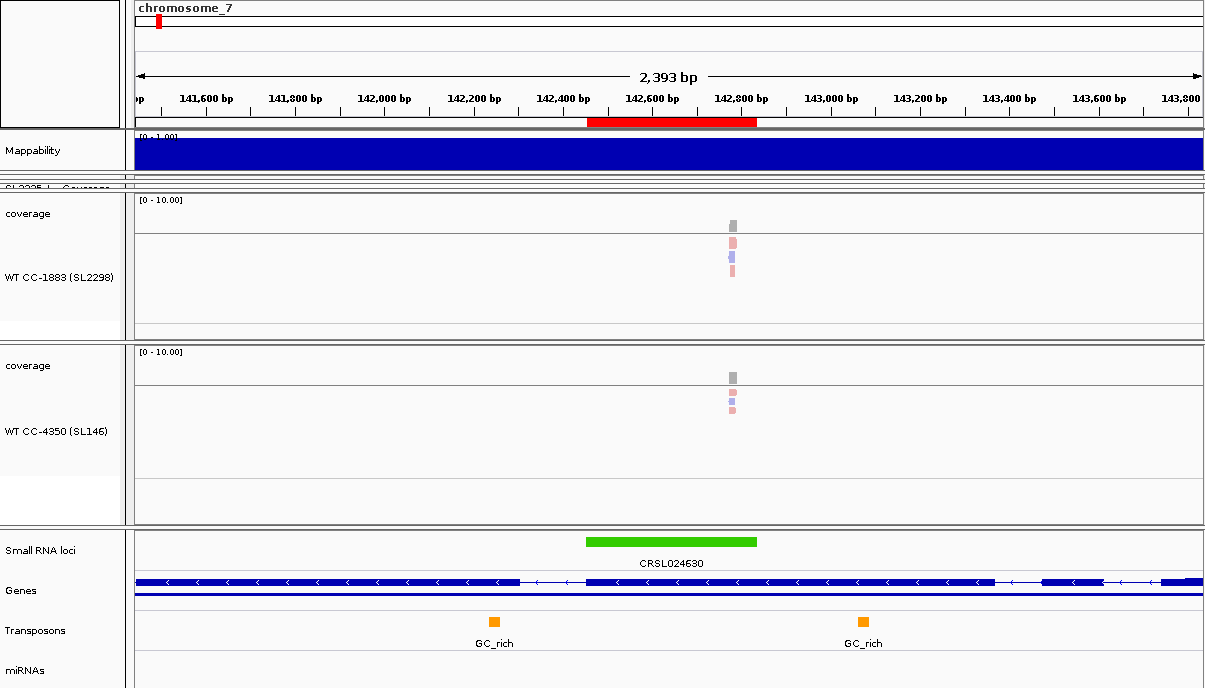

Supplement: S20 Fig — Tracks are annotated as in Fig 2. (PNG) [file pone.0242516.s020.png]
