# Supplementary material for: The small RNA locus map for Chlamydomonas reinhardtii
Source: PLoS One. 2020 Nov 19;15(11):e0242516. doi: 10.1371/journal.pone.0242516 (PMC7676726; doi:10.1371/journal.pone.0242516)
Supplement: Attachment — Submitted filename: 108108_1_rebuttal_1845878_qfpwww.pdf [file pone.0242516.s029.pdf]

We would like to express our utmost gratitude to the three anonymous reviewers for their constructive and insightful comments on our manuscript. We broadly agree with all comments made and have uploaded a preliminary revised version with changes highlighted in bold. We now deal with each of the reviewer comments in turn.

Reviewer #1

1. *L50-52: Can you predict where the unmapped read came from? Could viral infections be the source as in land plants?*

Having done a crude examination of unmapped reads, we couldn't find compelling evidence of them being of viral origin. The unmapped fraction in fact was in the same region as seen for other sRNA libraries in our lab which we found to occur for a number of reasons such as sequencing errors, incomplete assembly, differences between the sequenced lines and the reference line. Those all result in unmapped reads, which is also cause by since we employed a stringent mapping (0 mismatches).

2. *L67-68, which is the explanation?*

Thank you for querying this. After much closer inspection of the papers cited by Casas-Mollano et al. as evidence of the 23nt peak the evidence for the 23nt doesn't seem that strong and may even be a mistake on their part. Nonetheless, it is far from a critical piece of information for this paper and we have thus decided to remove this sentence.

3. *Fig 1D the reference to the A,C,G,U 5' should be re-positioned within Figure 1D panel space.*

Thanks, this has been addressed.

4. *Figure 3: it could be a supplementary figure based on the relevance given in the manuscript to this point.*

We agree, and have moved Fig3 to Supplement.

5. *P5, line 107: while commenting on strand bias there seems to be a mistake in strong bias definition, it should be x < 0.2 and x > 0.8, not "strong bias (0.2 < x < 0.8)", as in the text.*

Thank you for pointing this out, we have now corrected this error. We have duly corrected it in the text.

6. *P5, line 110: marked changes regarding locus size are not as striking in my opinion, in particular log size 6 and following, which is not marked in the graph (the cut off between 6 and 8). Maybe this curve should be split into two distribution graphs based on some important features (as repetitiveness?) that might allow a better definition of cut-offs.*

Thank you for pointing this out. You are correct that the changes in the density distribution are not as striking for locus size. A great deal of deliberation on our part went into deciding what to do about this. In the end, we decided that for the size classes there was benefit in having several different classes with the understanding that having additional potentially redundant cut-offs would not adversely effect the analysis. In doing this, we were partially driven by the albeit subtle changes in the curve, but also by the desire to have size classes that were biologically relevant and informative. For example, a locus <100nt might correspond to a single sRNA, while the 100-400nt size class covers the mean lengths of an exon (190nt) and an intron (373nt). There was a clear change in the graph at 1500nt and

then >3000nt captures the long tail. However, we neglected to fully explain these subtleties in our decision-making, something we have now rectified through some added explanation in the text. These choices were validated by the way size classes are differentially associated with different locus clusters in Figure 8.

7. *Fig 5: the legend has the C subfigure twice, the second should be D.*

Thank you for highlighting this. It has now been corrected.

8. *Table 1: I believe the data would be better presented in a plot, potentially something similar to the plot in Figure 1 A and B. The numbers are already presented in the supplementary spreadsheet.*

Thanks for pointing this out. We agree with this suggestion and have replaced Table 1 with a Figure (Fig 5) which is indeed a better way to present those results.

9. *Fig 6A: The boxplots regarding Stability of the clusters should be better described. What exactly does the y-axis in each "small plot" represent?*

Thank you for pointing this out, we understand that this isn't clear at the moment. Briefly, for this analysis we performed the clustering multiple times each time with a random sample of the loci (with replacement) of the same size as the original dataset. We then calculated the proportion of loci that retained their original clustering. We have clarified this in the figure legend and also elaborated on the approach in the methods section to ensure that it is better described.

10. *P6, line 142: analyses of stability and variance shows 7 as the optimal k, while gap statistics and NMI suggested 6 as the optimal. It is not clear why 6 was preferred. The MCA section in Methods is unclear regarding this point too.*

Thank you for querying this. The process of choosing the appropriate value of k is a complicated one and we appreciate that the explanation could be clearer. After your comment we re-visited our decision-making process and were reassured that a k value of 6 rather than 7 was indeed appropriate. The stability plots in Fig. 6A start with k=2 and it can be clearly seen for k=6 that stability is comparatively high for dimensions 7-10. Indeed, k values of 2,3 and 6 seem to be the only feasible values. k=7 is fairly unstable for all dimensions from 1-8. We have done some rewording of the methods to hopefully make this clearer.

11. *Fig S2-S5: please check legends, they are identical, although they should cover examples of loci in LC2 through LC5. These figures are not cited in the text, only S1 and S2.*

Thanks for pointing this out. This is now corrected and we have referenced all figures in the main text.

12. *Fig 9: I suggest using different colors in density plots to ease interpretation. LC tracks could share a color and Gene, TEs, DNA meth, and All loci should have a different color each.*

A good suggestion - this has been replotted with different colours.

13. *Supplementary Files S1: The full-annotated locus map should be provided as a spreadsheet file or as a text (.csv) file, not as a pdf file.*

Thanks for pointing this out. We originally submitted this file as a gff format. We are not sure why this got converted. We will make sure this is going to be in appropriate format in the final form, especially having suffered from the pains of pdf tables ourselves in the past.

14. *I may be misunderstanding Fig. 6E, but it looks strange that the observed sum-of-squares is smooth, but the expected is not. Is it possible that the in-figure reference is inverted?*

Indeed, the colours were inverted. Thanks a lot for that spot, we have now swapped them around.

Reviewer #2

1. *I am concerned that the methodology used does not adequately distinguish small RNA loci that are attributable to random RNA degradation products from loci that are truly fit the DCL / AGO paradigm. I think this is critical to maximize the utility of the annotations for the community. This issue was not directly addressed in the current version of the manuscript. There is cause for concern: 64% of the annotations overlap with protein-coding genes (lines 116-117), 55% with exons (line 118), and 41% of loci show strong strand bias (lines 123-124). These are all associations expected for breakdown products of mRNAs. Furthermore, only 11% of the loci were found to be dependent on CrDCL3 (line 123). Small RNA sequencing data from the other 2 DCL mutants are not yet available (line 211). One way that has been effective in angiosperms is to track the proportion of "DCL-sized" RNAs within all RNAs from each locus. Loci comprised of random degradation products will be single-stranded, generally touching exons, and have a very wide size distribution. In contrast, loci where the small RNAs are truly created by a DCL protein will have a very narrow size distribution. In any event, I think a strong effort to identify and flag small RNA loci that are less likely to be DCL / AGO silencing RNAs, and more likely to be degradation products, would be an important change to this study.*

Thank you for this very insightful comment which has helped us to reflect on the methodological approach. While it is likely that there are some RNA breakdown products picked-up in the sRNA sequencing, we do not think that the locus-map as a whole is undermined by this. For example, 54% of loci have a predominance for 21-nt sRNAs and 18% for 20-nt sRNAs, so the majority of sRNA loci do have a predominance for a specific RNA size.

However, your point does raise a very valid concern with implications for the interpretation of LC4. Although we posit some explanations for these loci (e.g. DCL-mediated sRNA production without an accessory protein to provide PAZ domain-like sRNA measurement), given the very strong strand bias and association with genic regions we do agree that there is a risk that these loci predominantly represent degradation fragments. Therefore, we have now reworded how we discuss LC4 in the discussion to reflect this. This also reveals a key advantage of the clustering approach in that should LC4 indead represent degradation products, they have been successfully grouped together into a seperate cluster such that they don't undermine the insights gained from the other locus clusters.

2. *One of the key results likely to be used by others is the final GFF3 file (Sup File S1). The Description fields in this file are extremely verbose. Do these load well on a genome browser? I suggest it might be good to store most of the information currently in the Description field in a separate flat file, and limit the GFF3 descriptions to key information (locus name, the LC group).*

Thank you for pointing this out. In a pursuit to share as many details as possible, we appreciate that this can be too verbose, as righlfully noticed here. In order to not compromise detail too much, we have created a second, toned down, version as csv which now includes essential details such as name, position and LC. As for the gff, we kept all details in since it loads quickly in a genome browser, but also into other tools such R in which those feature can be used as efficient filters.

3. *Sup Table S1 would be much more useful for future researchers if it had a column with the direct accession numbers for the raw sequencing libraries.*

We have included another table which includes direct accession number for ENA as well as numerous other meta data in Sup Table S6 i.e. "Supp_Table_S6_library_ENA_accession"

4. *Figures showing genome browser snapshots are too small; the text is mostly illegible on screen and when printed. This includes Figure 4 and Figures S1-S5.*

The snapshots have been improved to ensure better readability.

5. *Lines 67-68: This is unclear to me. Did the authors do Northerns? Please clarify / re-write.*

Thank you for querying this. After much closer inspection of the papers cited by Casas-Mollano et al. as evidence of the 23nt peak the evidence for the 23nt doesn't seem that strong and may even be a mistake on their part. Nonetheless, it is far from a critical piece of information for this paper and we have thus decided to remove this sentence.

6. *Figure 2B: X-axis label, perhaps change to "number of reads in library" for clarity.*

We agree and have changed it accordingly

7. *Figure 4 caption: The acronym "CRSL" should be defined.*

CRSL is now been duly defined in the manuscript

8. *Line 387: Reference #29 (line 509): There is not enough information here to find the data.*

We have used the appropriate bibtex code to reference this Zenodo share (https://zenodo.org/record/3862405/export/hx). The current cite format does somehow omit some information. We hope this will be fixed by the publisher but we have also provided the full DOI address in the “additional information” section just in-case. We will keep an eye on how it comes out.

9. *Style suggestion on title: What is "secret" about the genome? I didn't really understand that first part of the title. Perhaps consider revision to make it more factual and less literary. Just "A small RNA locus map for Chlamydomonas reinhardtii"?*

Thank you for this suggestion, we have adapted the title to make it more descriptive.

Reviewer #3

1. *…the evolutionary implications are not clear. The authors state in the abstract that "These results are consistent with the idea that there was diversification in sRNA mechanisms after the evolutionary divergence of algae from higher plant lineages." Although in the end this may prove to be correct, the only species compared are Arabidopsis thaliana (as representative of land plants) and Chlamydomonas*

reinhardtii (as representative of green algae). With this very limited information it is not possible to infer the sRNA loci (much less sRNA mechanisms) in an ancestral species. It remains formally possible that an ancestral progenitor species had a greater diversity of sRNA loci that were subsequently lost in a selective manner in specific lineages. Moreover, the diversity of sRNA loci may not correlate strictly with the diversity of the RNAi machinery since, at least some loci, do not appear to be associated with RNAi components such as Dicer or Argonaute.

Thank you for these insightful comments. As we followed a very similar methodological approach to that used to produce the Arabidopsis sRNA locus map published in Hardcastle et al. (2018), we wanted to take the opportunity to compare the results and build upon the ongoing discussion concerning the evolution of sRNA mechanisms in Chlamydomonas (e.g. Valli et al. 2016). Your point about the possibility of an ancestral progenitor with greater diversity that was then lost is very valid. You are also of course correct about the limitations to what can be concluded from this study and the limited comparisons that can be made. We see our approach as a useful tool for hypothesis generation which can be complemented by more in-depth exploration in the future. With this in mind, and taking on board your comments, we have elaborated on our discussion of the evolutionary implications of our study, which we hope now gives a more balanced account.

2. *I may have missed it but I could not find a table listing the specific sRNA loci assigned to each of the locus classes. It would be very useful to provide the class annotation of each sRNA locus in order to facilitate future analyses of sRNA biogenesis and function.*

That information was indeed missing, thanks for bringing it up. We have now included this in the gff file (column LC) as well as in another cleaner table (Supp_Table_S7_loci_class_annotation).

3. *Figures S2 to S5 have the same legend but they correspond to different loci. It would be useful to provide for each locus class, as supplementary figures, two examples of typical sRNA loci.*

Thanks for pointing this out, this was an error on our part, the captions have now been corrected. Unfortunately, due to the ongoing pandemic-related restrictions we were unable to run to get a genome browser session to run to this point to create more loci figures.

4. *If information is available, the paper would be strengthened by some locus class validation based on features not used to generate the classification.*

Thank you for this suggestion. In fact, not all annotation features were used predictively in the MCA and clustering process, and so these "supplementary" annotations as outlined in supplementary table S3 can provide some cross-validation. With that in mind, we have now included an additional heatmap as a supplementary figure which shows associations for some of these supplementary annotations as well as corresponding explanations in the text. Further validation is provided by the chromosome tracks in figure 9 showing the distinct genomic distributions of each locus cluster despite chromosomal location not being a factor in the clustering.

5. *Pg 5, line 108. I think you mean "strong bias (0.2 > x > 0.8)."*

Thank you for pointing this out, we have now corrected this error.

6. *Pg 7, Table 1. Some of the annotation features are obvious but some abbreviations may need clarification using footnotes.*

Table 1 has been replaced by the new Fig 5, annotation/abbreviations should now be more obvious.

7. *Pg 8, lines 156-157. This sentence is not clear. Additionally, the legends to Figures S2-S5 do not refer to LC2 paragon (CSRL003890).*

Thank you for pointing this out. We have now moved the reference to the paragons to earlier in the section where we introduce the six clusters. We hope this is now clearer.
